# Diagnostic value of biochemical markers (NashTest) for the prediction of non alcoholo steato hepatitis in patients with non-alcoholic fatty liver disease

**DOI:** 10.1186/1471-230X-6-34

**Published:** 2006-11-10

**Authors:** Thierry Poynard, Vlad Ratziu, Frederic Charlotte, Djamila Messous, Mona Munteanu, Françoise Imbert-Bismut, Julien Massard, Luninita Bonyhay, Mohamed Tahiri, Dominique Thabut, Jean François Cadranel, Brigitte Le Bail, Victor de Ledinghen, the LIDO Study Group and the CYTOL study group

**Affiliations:** 1 Hepato-Gastroenterology, AP-HP Groupe Hospitalier Pitié-Salpêtrière, Paris, France; 2 Pathology, AP-HP Groupe Hospitalier Pitié-Salpêtrière, Paris, France; 3 Biochemistry AP-HP Groupe Hospitalier Pitié-Salpêtrière, Paris, France; 4 Biopredictive, Paris, France; 5 Hepato-Gastroenterology, Hôpital Creil, Creil, France; 6 Pathology, Hôpital Haut Lévèque, Bordeaux, France; 7 Hepato-Gastroenterology, Hôpital Haut Lévèque, Bordeaux, France

## Abstract

**Background:**

Liver biopsy is considered the gold standard for assessing histologic lesions of non-alcoholic fatty liver disease (NAFLD). The aim was to develop and validate a new biomarker of non alcoholic steato hepatitis (NASH) the NashTest (NT) in patients with NAFLD.

**Methods:**

160 patients with NAFLD were prospectively included in a training group, 97 were included in a multicenter validation group and 383 controls. Histological diagnoses used Kleiner et al's scoring system, with 3 classes for NASH: "Not NASH", "Borderline", "NASH"). The area under the ROC curves (AUROC), sensitivity (Se), specificity (Sp), and positive and negative predictive values (PPV, NPV) were assessed.

**Results:**

NT was developed using patented algorithms combining 13 parameters: age, sex, height, weight, and serum levels of triglycerides, cholesterol, alpha2macroglobulin, apolipoprotein A1, haptoglobin, gamma-glutamyl-transpeptidase, transaminases ALT, AST, and total bilirubin. AUROCs of NT for the diagnosis of NASH in the training and validation groups were, respectively, 0.79 (95%CI 0.69–0.86) and 0.79 (95%CI 0.67–0.87; P = 0.94); for the diagnosis of borderline NASH they were: 0.69 (95%CI 0.60–0.77) and 0.69 (95%CI 0.57–0.78; P = 0.98) and for the diagnosis of no NASH, 0.77 (95%CI 0.68–0.84) and 0.83 (95%CI 0.67–0.90; P = 0.34). When the two groups were pooled together the NashTest Sp for NASH = 94% (PPV = 66%), and Se = 33% (NPV = 81%); for borderline NASH or NASH Sp = 50% (PPV = 74%) and Se = 88% (NPV = 72%).

**Conclusion**

In patients with non-alcoholic fatty liver disease, NashTest, a simple and non-invasive biomarker reliably predicts the presence or absence of NASH.

## Background

Non-alcoholic fatty liver disease (NAFLD) represents a spectrum of conditions characterized histologically by an excessive accumulation of hepatic fat in the absence of alcohol consumption. Two main histological patterns of NAFLD have been described: bland steatosis and steatohepatitis (NASH). NAFLD is an increasingly recognized cause of liver-related morbidity and mortality [1-3]. Although the majority of patients do not develop complications, 28% may develop serious liver sequelae, including end-stage liver disease and hepatocellular carcinoma. Those at highest risk include patients with significant hepatic necro-inflammation and fibrosis [1-6]. Liver biopsy, therefore, has been recommended for confirming its diagnosis and for providing prognostic information [7].

There are several drawbacks in using liver biopsy for this purpose [8]. It is an invasive and costly procedure, and is prone to complications, some minor, such as pain, others more severe with a recorded risk of death of 0.01% [9-11]. Notably, as in other chronic liver diseases, there is high sampling variability; and high intra- and inter-pathologist variability [12-14]. Most importantly, the number of patients at risk for NAFLD is high enough that liver biopsy is not a practical and efficient tool for identifying those at risk of NASH and advanced fibrosis. Indeed an estimated 15 to 20% of the Western European population has steatosis [15], while more than half of Americans are overweight or obese.

Because liver biopsy is impossible to perform in such large cohorts of individuals, some investigators have tried to identify simple non-invasive markers of liver injury in patients with NAFLD.

Different studies have shown that factors which are associated with NASH in patients with NAFLD are male gender, age, the extent of obesity, type 2 diabetes, high levels of alanine aminotransferase, aspartate aminotransferase and triglycerides, high HOMA indices of insulin resistance, systemic hypertension, high levels of C-peptide [6,16-21], hyaluronic acid and type VI collagen [22], TNF-alpha and IL-8 [23], and serum acute phase proteins [24]. However, these findings are not consistent between studies and have been generated through retrospective studies, all amenable to known and unknown biases.

In the last five years, we have developed several panels of simple biochemical markers known as FibroTest (FT), ActiTest, (Biopredictive Paris, France,) SteatoTest (ST) (Biopredictive Paris, France) and AshTest (Biopredictive Paris, France) (HCV/HBV FibroSURE, Steato-FibroSURE, Ash-FibroSURE in the US). ActiTest was developed for the grading of necroinflammation in viral hepatitis C and B. AshTest was developed for the diagnosis of alcoholic-steato-hepatitis in heavy drinkers

FT has demonstrated high predictive values for advanced fibrosis in patients with NAFLD [25] similar to those previously observed for chronic hepatitis C [26-28], chronic hepatitis B [29,30], and alcoholic liver disease (ALD) [31,32]. The diagnostic value of FT was also confirmed in these different chronic liver diseases by independent groups and comparison with the other panels, glycomics and elastometry [32-34].

ST has demonstrated high predictive values for the diagnosis of steatosis in patients with NAFLD, chronic hepatitis C, chronic hepatitis B, and ALD [35].

AshTest has demonstrated high predictive values for the diagnosis of alcoholic steato-hepatitis in heavy drinkers [36].

Therefore NASH was the only important histological feature for which no biomarkers were available. The objective of the current study was to validate the diagnostic utility of a new panel of biomarkers, NashTest (NT), for the detection of NASH in patients with NAFLD in order to reduce the need for liver biopsy.

## Methods

### Study population

The populations screened for inclusion in the present study were the same as those used in previously published validation studies for FT in NAFLD. The inclusion criteria were the same for the validation groups. For the training groups, the only difference in the present study was the exclusion of patients without histological steatosis, though these have been included in FT training and validation studies [25]. The rational of excluding patients without steatosis was to focus on the diagnosis of NASH versus no NASH among patients with non alcoholic steatosis. This has been possible since the validation of a non-invasive test for the diagnosis of steatosis recently published [35].

#### Training group

The inclusion criteria were patients with suspected NAFLD hospitalized in our department having steatosis at liver biopsy. Exclusion criteria included a daily alcohol consumption of at least 50 gm of pure ethanol equivalent for males and 30 gm for females during the preceding year, concomitant liver diseases (the presence of HCV antibody or HBs antigen, auto-immune hepatitis, hemochromatosis diagnosed by genetic markers, Wilson's disease, alpha anti-trypsine deficiency), HIV antibodies and immunosuppression, and an interval greater than 3 months between serum sampling and liver biopsy. Between January 2001 and December 2004, 238 patients were hospitalized for suspected NAFLD; 160 patients were included and 78 patients were excluded for the following reasons (several causes were present in four patients): no steatosis in 20, associated liver disease in two, missing data in 39 (biomarkers not performed in 37 patients, biopsy not performed in two patients), and an interval between biopsy and markers greater than 3 months in 21 patients. Characteristics are given in Table 1.

#### Validation group

These were patients from a prospective multicentric study (CYTOL study). The aim of the CYTOL study was to assess the cause of chronic abnormal ALT or GGT values in patients without heavy alcohol consumption, who had no markers of HCV (HCV antibody), HBV (HBs antigen), autoimmune hepatitis (negative for anti-actin, anti-nuclear, anti-LKM1 antibodies), hemochromatosis (genetic markers), Wilson's disease, or alpha anti-trypsine deficiency. For the present study only the CYTOL patients with hepatic steatosis at biopsy with suspicion of NAFLD were considered for inclusion. Between February 2002 and August 2004, among the 274 patients of the CYTOL study, 166 patients with steatosis at biopsy were considered for inclusion, 97 patients were included and 69 patients were excluded for the following reasons: 31 because they were being followed in the training center (but not included in the training set), and 38 due to the presence of miscellaneous associated liver diseases. Characteristics are given in Table 1.

#### Control group

A total of 383 prospectively included blood donors or healthy volunteers from the training enter were used as controls.

This protocol was carried out in compliance with the Helsinki Declaration. Serum sampling and liver biopsy were part of the routine in the different institutions.All patients and controls gave verbal informed consent for the use of data and serum for research purposes and this was approved by the ethical committee of Paris Pitié Salpêtrière Hospital.

### Histological analysis

Liver biopsies were fixed, paraffin-embedded, and stained with at least hematoxylin-eosin-safran, iron staining, and Masson's trichrome or picrosirius red for collagen. A single pathologist, unaware of patient characteristics, analyzed the histological features in each group, FC in the training group and BLB in the validation group.

A scoring system recently published by Kleiner et al [14] was used. Fibrosis was staged as follows: stage 0 = no fibrosis; stage 1 = perisinusoidal or periportal fibrosis with 3 different patterns: 1A = mild, zone 3, perisinusoidal; 1B = moderate, zone 3, perisinusoidal fibrosis, and 1C = portal/periportal fibrosis; stage 2 = perisinusoidal and portal/periportal fibrosis; stage 3 = bridging fibrosis; stage 4 = cirrhosis. Steatosis was scored from 0 to 3 with a four grades scoring system from S0 to S3: S0_no steatosis or less than 5% (low to medium -power evaluation of parenchymal involvement by steatosis), S1_5%–33%, S2_ > 33%–66%, S3_ > 66%.

We used two definitions of NASH. The main endpoint was steatohepatitis defined as a NAS score of 5 or greater. The histological NASH score (NAS) is defined as the unweighted sum of the scores for steatosis (0–3), lobular inflammation (0–3), and ballooning (0–2); scores therefore ranged from 0 to 8. Cases with NAS of 0 to 2 were considered not diagnostic of NASH; on the other hand, cases with scores of 5 or greater were diagnosed as NASH. Cases with activity scores of 3 and 4 were considered as borderline (probable) NASH [14]. The second endpoint was the determination of the pathologist of whether the NASH is present or not.

### Serum biochemical markers

FibroTest (FT) (Biopredictive, Paris, France, patented artificial intelligence algorithm USPTO 6,631,330) includes total bilirubin, GGT, α_2_-macroglobulin, apolipoprotein A1, and haptoglobin, corrected for age and gender, and is designed for a quantitative assessment of fibrosis.

SteatoTest (ST) is a new panel (SteatoTest, Biopredictive, Paris, France, patent pending), recently published, combining the 6 components of the FibroTest-ActiTest adjusted for age, gender and BMI, plus AST, serum glucose, triglycerides and cholesterol. ST scores range from zero to 1.00, with higher scores indicating a greater probability of significant steatosis.

The new panel, NT (Biopredictive, Paris, France, patent pending), was constructed in the training group combining age, gender, the 6 components of the FibroTest-ActiTest (Biopredictive, Paris, France, patented artificial intelligence algorithm USPTO 6,631,330) plus weight, height, AST, serum glucose, triglycerides, cholesterol and ST. AST, ALT, GGT, cholesterol, triglycerides, and total bilirubin were measured by an autoanalyzer Hitachi 917 Automate (Mannheim, Germany) using Roche Diagnostics reagents (Mannheim, Germany). Alpha_2_-macroglobulin, apolipoprotein A1, and haptoglobin were measured using an automatic nephelometer (BNII, Dade Behring; Marburg, Germany). The laboratory followed the recommended and validated procedures to insure reproducibility between FT components [27,28]. All the biochemical components had been prospectively assessed and assays were performed on fresh serum. NT was computed only if ST demonstrated a steatosis. The first algorithm used the SteatoTest. If there is no presumed steatosis the result of NashTest is Non NASH. If there is a steatosis the other algorithms are computed. The next algorithms included all the components of the FibroTest and of the SteatoTest, using specific weights in three logistic regression formula, one for the diagnosis of no NASH, one for borderline NASH and one for the diagnostic of NASH. The most discriminant parameters were weight and gender for the clinical parameters, AST, GGT and glucose for biological parameters. The three regressions permitted to generate probabilities and to classify the patient in one of the 3 categories. All the parameters were also used to generate security algorithms to detect abnormal values as previously described (27)

### Statistical analyses

The primary outcome was the diagnosis of NASH in the three categories according to Kleiner et al: NASH, Borderline NASH, No NASH.

Sensitivity analysis compared patients without alcohol consumption to patients with a small alcohol consumption (less than 20 g a day for females and less than 30 g for males) and to those with mild consumption (between 20 and 30 g for females, between 30 to 50 g for males), patients with elevated or normal baseline ALTs; patients without a high risk of biochemical components failure, patients with a baseline biopsy length less or greater than 25 mm, and with or without fragmentation. The diagnostic value of NT was also estimated using the pathologist determination of Nash or no Nash.

Clinically significant discordance between NT and biopsy was defined as a two classification discordance: NASH as predicted by NT and no Nash as observed at biopsy; or the inverse, no Nash as predicted by NT and Nash as observed at biopsy. The cause of high discordance between NT and biopsy was attributed according to the respective risk factors of failure. Risk factors of NT failure were hemolysis, Gilbert's syndrome, acute inflammation, extrahepatic cholestasis and extreme values outside the 98% percentiles for one component of NT. Risk factors of biopsy failure were biopsy size (less than 25 mm) and fragmentation (more than one fragment). Failure attributable to biopsy (false negative) was suspected when the biopsy was smaller than 15 mm and fragmented, in the absence of risk factors of NT failure and with at least another sign of steato-hepatitis not belonging to Kleiner scoring system, such as piece meal necrosis.

Statistical analysis used Fisher's exact test, the chi-square test, Student's t test, the Mann-Whitney test, and variance analysis used the Bonferroni all-pair wise and Tukey-Kramer multiple-comparison tests to take into account the multiple comparisons and multiple logistic regression for multivariate analysis. The diagnostic values of the markers were assessed using sensitivities, specificities, positive (PPV) and negative predictive values (NPV), and the areas under the receiver operating characteristic curves (AUROC). AUROC curves were calculated including FT quantitative values using an empirical non-parametric method according to Delong et al [37] and compared using the method of Zhou et al [38]. For all analyses, two-sided statistical tests were used; a P-value of 0.05 or less was considered significant. Number Cruncher Statistical Systems 2003 software (NCSS, Kaysville, Utah, USA) was used for all analyses.

## Results

### Patients

A total of 160 patients were included in the training group and 97 in the validation group. Characteristics of included patients, as well as those of the non-included groups were similar (Table 1). The only significant differences observed were related to the inclusion criteria, with more metabolic risk factors, more steatosis and more advanced fibrosis in the included than the non-included patients (Table 1). When patients included in the training group were compared to those included in the validation group, those in the training group were found to be older, and to have more diabetes and arterial hypertension, more elevated weight and less NASH. The biopsy size in the training group was longer with more portal tracts than in the validation group (Table 1).

### Diagnosis of NASH

When compared to patients with no NASH, those with NASH or borderline NASH were older, had a higher weight, higher alpha2 macroglobulin, higher ALT and AST, lower GGT, higher glucose higher triglycerides, higher fibrosis stages as assessed by FibroTest and higher steatosis grades as assessed by SteatoTest (Table 2 and Table 3). In multivariate analysis the most discriminant parameters were weight, AST and GGT (Table 3).

When compared to patients with no or borderline NASH, those with NASH were older, had higher alpha2 macroglobulin, higher ALT and AST, higher glucose, higher triglycerides, higher fibrosis stages as assessed by FibroTest and higher steatosis grades as assessed by SteatoTest (Table 2 and Table 3). In multivariate analysis the most discriminant parameters were female gender and glucose (Table 3).

Diagnostic values of NT for predicting borderline NASH and NASH in different groups, are given in Table 4 for the concordance with biopsy results, and in Table 5 for sensitivity, specificity and predictive values. There were similar discordance rates and predictive values between training and validation groups. When the two groups were pooled together, the discordance rates were 4% for two classes (clinically significant) and 39% for one class, the NashTest Sp for Nash = 94% (PPV = 66%) and Se = 33% (NPV = 81%); for borderline Nash or Nash, Sp = 50% (PPV = 74%) and Se = 88% (NPV = 72%) (Table 5).

**Figure 1:**
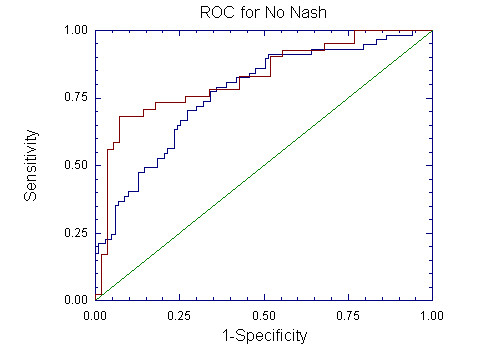
ROC curves of the NashTest for the diagnosis of No NASH in Training and in Validation Groups.

**Figure 2:**
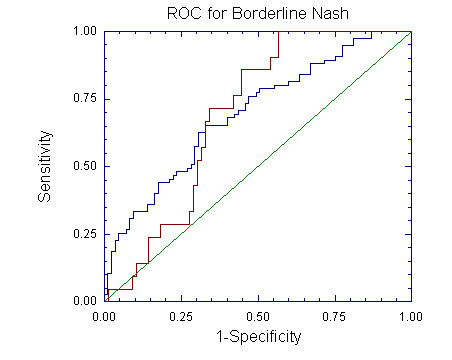
ROC curves of the NashTest for the diagnosis of Borderline NASH in Training and in Validation Groups.

**Figure 3:**
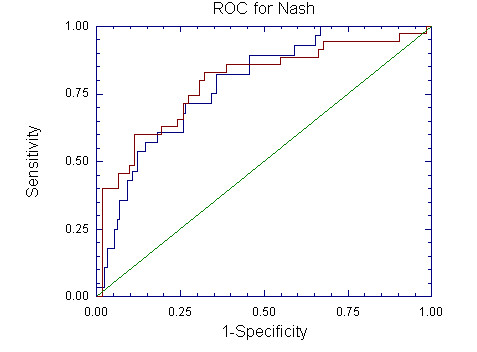
ROC curves of the NashTest for the diagnosis of NASH in Training and in Validation Groups.

ROC curves of NT for predicting NASH or borderline NASH are illustrated in Figures 1 to 3. There was no difference between the AUROCs in the training and validation groups, respectively, for No NASH [AUROC = 0.77 (95%CI 0.68–0.84) versus 0.83 (95%CI 0.67–0.90; P = 0.34)] (Figure 1); for Borderline NASH [AUROC = 0.69 (95%CI 0.60–0.77) versus 0.69 (95%CI 0.57–0.78; P = 0.98)] (Figure 2); or for NASH [AUROC = 0.79 (95%CI 0.69–0.86) versus 0.79 (95%CI 0.67–0.78; P = 0.87)] (Figure 3).

**Figure 4:**
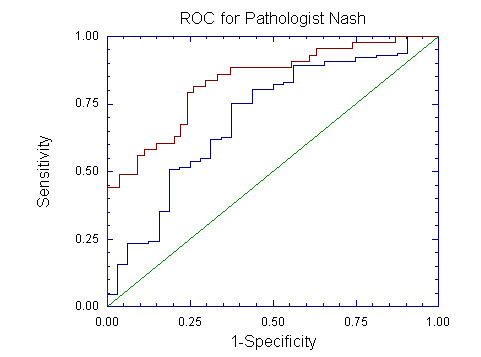
**ROC curves of the NashTest for the diagnosis of NASH made by pathologist in Training and in Validation Groups**. The diagonal line represents that achieved by chance alone (area under the curve 0.50); the ideal area under the curve is 1.00. Dotted curve is the Training Group, Solid curve is the Validation Group. There was no difference between the area under the ROC curves (AUROCs) for No NASH [AUROC = 0.77 (95%CI 0.68–0.84) versus 0.83 (95%CI 0.72–0.90; P = 0.34)], for Borderline NASH [AUROC = 0.69 (95%CI 0.60–0.77) versus 0.69 (95%CI 0.57–0.78; P = 0.98)] and for NASH [AUROC = 0.79 (95%CI 0.69–0.86) versus 0.79 (95%CI 0.67–0.78; P = 0.87)] in the Training and Validation Groups, respectively.

The AUROC of NT for the diagnosis of Nash determined by the pathologist (171 NASH and 86 Non NASH) independent of NAS was 0.78 (95% CI 0.71–0.83) on the overall population, without difference between the training and validation groups: 0.69 (95% CI 0.56–0.79) and 0.80 (95% CI 0.69–0.87) respectively (Figure 4).

**Figure 5:**
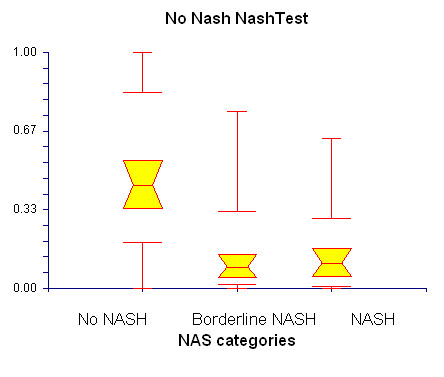
Value of the index No-NashTest designed for the diagnosis of No NASH.

**Figure 6:**
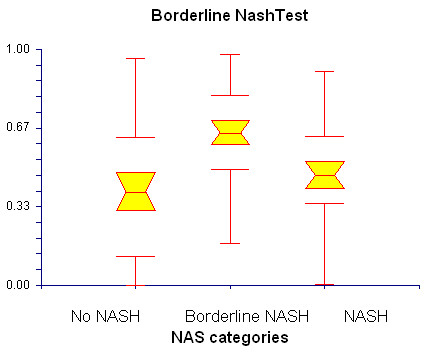
Value of the index Borderline-NashTest designed for the diagnosis of No NASH.

**Figure 7:**
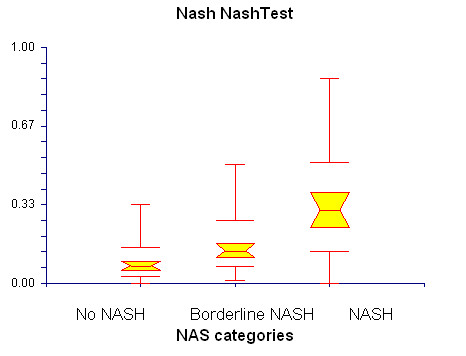
Value of the index Nash-NashTest designed for the diagnosis of NASH.

**Figure 8:**
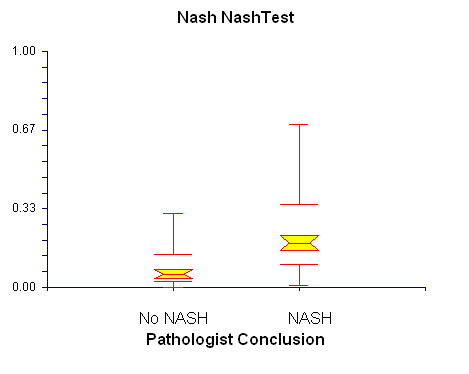
**Value of the index Nash-NashTest for the diagnostic of NASH as stated by the pathologist independent of NAS score**. Each index is a specific logistic regression combining parameters weighted according to their diagnostic values. Notched box plots showing the relationship between NAS category (No NASH n = 98), borderline NASH n = 96 and NASH n = 63 and index of No-Nash (Figure 4), index of Borderline-Nash (Figure 5) and index of Nash (Figure 6) and between the pathologist conclusion and index of Nash (Figure 7). The horizontal line inside each box represents the median, and the width of each box the median ± 1.57 interquartile range/√ n (to assess the 95% level of significance between group medians). Failure of the shaded boxes to overlap signifies statistical significance (P < 0.05). The horizontal lines above and below each box encompass the inter-quartile range (from 25^th ^to 75^th ^percentile), and the vertical lines from the ends of the box encompass the adjacent values (upper: 75^th ^percentile plus 1.5 times interquartile range, lower 25^th ^percentile minus 1.5 times interquartile range).

The values of the different components of NT are detailed in Figure 5 to 8: Figure 5 for the No-NashTest designed for the diagnosis of No NASH, Figure 6 for the Borderline-NashTest designed for the diagnosis of No NASH, Figure 7 for the Nash-NashTest designed for the diagnosis of NASH and Figure 8 for the Nash-NashTest for the diagnosis of NASH as defined by the pathologist.

#### Analysis of discordance

Among the 11 clinically significant discordances (two classes difference) observed, nine were attributable to NT failure (eight false negatives and one false positive) and two to biopsy (false negatives).

In the training group, there was a clinically significant discordance in three patients (2%), all with NT predicting NASH and biopsy showing no NASH. One of these cases had a good quality biopsy (30 mm non fragmented) but with piecemeal necrosis and no cause of NT false positive; two cases had a poor quality biopsy (15 mm and five fragments, 22 mm and two fragments) with piece-meal necrosis at biopsy and no cause of NT false positive.

In the validation group there was clinically significant discordance in eight patients (8%): all were predicted to have no NASH with NT and NASH at biopsy. None of these eight patients had a good quality biopsy. There was a very low triglyceride level (0.1 and 0.3 mmol/L) in two patients suggesting two instances of NT's false negatives.

#### Sensitivity analyses

Sensitivity analyses revealed that the NT AUROCs for the diagnosis of NASH (Table 6) and the diagnosis of borderline NASH or NASH (Table 7) were not affected by groups, ALT values, alcohol consumption, Gilbert's syndrome, acute inflammation, absence of steatosis, or biopsy sample length. AUROCs were higher, though not significantly, in patients with non-fragmented biopsies than in those with fragmented biopsies. (Tables 4 and 5). Only nine patients had ALT below the proposed new normal range for serum ALT and three of those had borderline NASH.

#### Controls

Among the 383 controls 26/383 (7%) had steatosis estimated by SteatoTest and none had NASH.

## Discussion

Mass screening for significant liver injury in patients with NAFLD will be an important medical challenge in the years to come due to the epidemics of obesity and diabetes. The inability of liver biopsy to meet this challenge makes the development of non-invasive, readily available and easy to perform serum markers a high priority. In these patients the priority is to estimate the severity of fibrosis but also to identify patients with steato-hepatitis among those with steatosis.

Many studies on non-invasive panels with significant diagnostic value for fibrosis have been published but so far the most studied biomarker is the FT [39], having a specific validation in NAFLD [25]. Many previous studies have highlighted the potential utility of FT for patients infected with HCV [26-28,32-34], HBV [29,30] and patients with ALD [31,32,34].

Few tests have yet been developed for the diagnosis of steatosis and steato hepatitis [17-21]. We recently highlighted the potential utility of ST for the prediction of steatosis in patients with NAFLD, as well as in patients infected with HCV, HBV and ALD [35]. Since the validation of ST it is therefore possible to focus on the diagnosis of NASH in patient with NAFLD after exclusion of patients without steatosis. The algorithm of NT excluded patients with steatosis predicted by ST. When screened patients without histological steatosis were included in a sensitivity analysis the diagnostic value of NT was not different than in patients with steatosis only.

We also recently demonstrated the potential utility of AshTest for the prediction of alcoholic steato-hepatitis in heavy drinkers [36]. AshTest was designed for the diagnosis of patients with severe alcoholic steato hepatitis needing specific treatment [36] and does not have significant diagnostic value for the diagnosis of NASH (data not shown). Therefore a specific test for NASH was necessary to complete the non invasive estimation of frequent histological features in patients with NAFLD.

The most significant components of NT were the metabolic factors (mostly weight, triglycerides and glucose), as previously observed [17-21], but also A2M and apoA1. These proteins have been proven to be associated with fibrosis [26] but also with steatosis [35], steato-hepatitis [24,25] and insulin resistance pathways [25]. A2M is a protease inhibitor but also has multiple functions as a binding, carrier and targeting protein [40]. In patients with NAFLD we previously demonstrated a very significant association between A2M and insulin levels, a hallmark of insulin resistance [25]. Other studies have observed an increase of A2M in diabetic patients [41]. Insulin is covalently bound to A2M [42] in plasma and A2M is a binding protein of Insulin-like Growth Factor Binding Protein-1 (IGFBP-1) which modifies the IGFBP-1/IGF interaction [43]. Therefore A2M can be directly involved both in the hepatic mechanisms of insulin resistance and fibrogenesis.

Comparisons with biomarkers of alcoholic steato hepatitis (ASH) [36] are important as ASH and NASH share many physio-pathological mechanisms and histological features. The same associations were observed for proteins in univariate analysis with a decrease in ApoA1, haptoglobin and an increase of A2M in NASH. However the decrease of apolipoprotein A1 in NASH was much lower than in patients with ASH. In our NASH population there was a much lower prevalence of cirrhosis, as well as severe steato-hepatitis in comparison with the population of ASH [36]. In the present NAFLD population, only two patients had a polymorphonuclear infiltrate (1.2%). In the first case there was a dramatic decrease in ApoA1 (0.05 g/L), as observed in alcoholic steato-hepatitis. In the second case the absolute value of ApoA1 was not decreased (1.72 g/L) but was relatively low in comparison with HDL cholesterol (1.64 mmol/L).

The present study has several limitations. First, the variability of the end point, histological steato-hepatitis, is even greater than for the other features of chronic liver disease, fibrosis and steatosis. There is both a significant limitation of liver biopsy due to its sampling variability [12], as observed for HCV hepatitis [44], and a high intra- and inter- observer variability [13,14]. In the present study only 25% (63/257) of biopsy samples reached the 25 mm minimum recommended by Bedossa et al for HCV [44]. When we used sensitivity analyses to compare the AUROCs of NT according to biopsy quality, there was no significant difference, although there was a trend in favor of better AUROCs with non-fragmented biopsies (Table 4). To reduce the observer variability related to the NASH definition, we used the recent NAS scoring system recommended by Kleiner et al [14]. To the extent that the NAS represents the severity of current liver injury, the proposed NT may separate those with more severe injury from those with little injury. This would be of great value in clinical trial situations where the investigator might want to enroll those with severe disease first or perhaps for identifying patients at greatest risk for progression. However the NAS was intended for use in monitoring changes in liver disease and other clinical situations, and was not intended to replace the pathologist's determination of whether NASH is present or not. Therefore we checked the utility of the NT for identifying patients with bona fide NASH using the pathologist determination. Indeed the value of NT for this diagnostic of NASH was fair (AUROC = 0.80 in the validation group).

Because of the biopsy variability, discordances between biomarker and biopsy results must be discussed case by case before attributing the cause of error to biomarkers or to biopsy. In the present study, 3.5 % of patients with discordance results were attributable to NT failure versus 0.5% to biopsy failure. Being a serum marker, NT has the advantage of giving a more global estimate of liver steato-hepatitis throughout the whole liver.

The first validation group included patients from a tertiary care center, which makes it liable to referral selection bias, but the second validation group was most representative of less specialized centers.

We have used less limited inclusion criteria concerning alcohol consumption with inclusion of patients consuming up to 49 g of alcohol per day, due to our national high consumption. There was no consensual limit. However when males consuming 30 g or women 20 g or more per day were excluded (only a total of 12 patients) according to recent guidelines for the diagnosis of NAFLD [45], the diagnostic value of NT was not significantly changed (Table 6 and Table 7). The prevalence of patients with metabolic risk factors and moderate alcohol consumption is important in many countries and should be also analyzed in diagnostic studies.

Another drawback of liver biopsy is that for most practitioners it seems almost unethical for it to be performed in patients with normal serum transaminases values. Unfortunately, many patients with NAFLD or NASH have normal ALT levels and some of them have advanced liver fibrosis [46-48]. In the present study 50% of patients with histological borderline NASH or NASH had ALT lower than 50 IU/L. NT AUROCs for the diagnosis of NASH or borderline NASH in NAFLD were unchanged in patients with ALT values lower than 50 IU/L (Table 6 and Table 7); therefore NT could be used to diagnose NASH even in patients that are not eligible for liver biopsy.

Although there is no specific treatment currently approved to treat liver injury in NAFLD, many are being developed. The diagnosis of advanced fibrosis or NASH could be very important for motivating patients to make diet or lifestyle modifications, for the intensive treatment of complications of the metabolic syndrome or for providing weight in favor of anti-obesity surgery. The early detection of advanced fibrosis or NASH is the first step reducing future cirrhosis-related deaths. Diagnosing silent cirrhosis has important consequences in terms of screening for portal hypertension and hepatocellular carcinoma, of preventing complications and of providing a timely indication for liver transplantation.

## Conclusion

Among patients with suspected NAFLD, the new generation of biomarkers such as FT, ST and NT will allow better identification of those at risk and reassurance for patients without fibrosis or NASH. Biomarkers as a first-line estimate of injury in chronic liver diseases should reduce the need for liver biopsy [49].

## Abbreviations

A2M, alpha2macroglobulin, GGT, γ-glutamyl-transpeptidase; ALT, alanine aminotransferase; ROC, receiver operating characteristic; AST, aspartate aminotransferase; NPV, negative predictive value; PPV, positive predictive value; ULN, upper limit of normal.

## Competing interests

TP is the inventor of the patented tests (Fibrotest, ActiTest, SteatoTest, NashTest and AshTest), is a consultant, and has a capital interest in Biopredictive, the company marketing these tests. The royalties of these tests belong to Assistance Publique Hôpitaux de Paris. MM is a full employee of Biopredictive.

## Authors' contributions

TP conceived the study and its design, participated in the coordination, performed the statistical analysis and wrote the article. VR participated in its design, in the liver biopsies and coordination. FC and BLB carried out histological analyses. DJ and FIB carried out the biochemical analyses. MM participated in the statistical analysis. JM, LB, MT,DT, JFC, VdL participated in the management of patients and coordination. All authors read and approved the final manuscript.

## Pre-publication history

The pre-publication history for this paper can be accessed here:



## Figures and Tables

**Table 1:** Characteristics of included or non-included patients

	**Training Group**			**Validation Group**			**Significance p training vs validation included**
**Characteristic**	**Included N = 160**	**Non-included N = 78**	**Significance p**	**Included N = 97**	**Non-included N = 69**	**Significance p**	

**Demographics***mean (se)or n (%)*							
Age at biopsy, *years*	52.9 (0.89)	54.2 (1.37)	0.34	48.5 (1.3)	49.1 (1.5)	0.72	0.01
Male	96 (60)	47 (60)	0.36	57 (59)	38 (69)	0.64	0.84
Height, *meter*	1.68 (0.01)	1.66 (0.01)	0.20	1.69 (0.01)	1.67 (0.01)	0.27	0.81
Weight, *kg*	83.6 (1.2)	76.3 (2.6)	0.01	77.4 (1.5)	74.0 (1.9)	0.04	0.002
Body Mass Index, *kg/m2*	29.6 (0.4)	27.6 (0.9)	0.03	27.1 (0.5)	26.4 (0.6)	0.11	< 0.0001
**Metabolic factor (%)**							
Diabetes	65/160 (41)	12/78 (15)	< 0.0001	31/97 (32)	15/69 (22)	0.62	0.0002
Arterial Hypertension	53/160 (33)	6/23 (26)		15/97 (16)	16/69 (23)	0.21	0.002
Body Mass Index greater than 27 kg/m^2^	101/160 (63)	16/34 (47)		43/97 (44)	22/69 (32)	0.11	0.003
Triglycerides greater than 1.5	70/160 (44)	20/54 (37)		34/97 (35)	15/69 (22)	0.06	0.17
HDL cholesterol lower than 40 mg/L	36/151 (24)	6/38 (16)		15/94 (16)	15/66 (23)	0.28	0.14
Metabolic factor							
None	1/160 (1)	22/78 (28)		2/97 (2)	0/69	0.23	0.30
At least one	159/160 (99)	56/78 (72)		95/97 (98)	69/69		
**Alcohol consumption**, *n (%)*							
No	114/160 (71)	61/78 (78)	0.25	57/97 (59)	41/69 (59)		
Yes	46/160 (29)	17/78 (22)		40/97 (41)	28/69 (41)	0.63	0.04
**Fibrosis stage**, *n (%)*							
*Non advanced fibrosis*	*121/160 (76)*	*71/75 (95)*	0.0001	*66/97(68)*	*51/69(74)*	*0.48*	*0.12*
No fibrosis	69/160 (41)	50/75 (67)		25/97 (27)	21/69 (30)		
Fibrosis perisinusoidal or periportal	52/160 (33)	21/75 (28)		41/97 (41)	30/69 (43)		
A: mild, zone 3, perisinusoidal	29/160 (18)	19/75 (25)		16/97 (19)	18/69 (25)		
B: moderate, zone 3, perisinusoidal	1/160 (1)	2/75 (3)		15/97 (15)	0/69 (0)		
C: portal/periportal	22/160 (14)	0/75 (0)		10/97 (22)	12/69 (18)		
*Advanced Fibrosis*	*39/160 (24)*	*4/75 (3)*		*31/97 (32)*	*18/69(26)*		
Perisinusoidal and portal/periportal	20/160 (13)	1/75(1)		15/97 (15)	13/69 (19)		
Bridging	10/160 (6)	1/75 (1)		12/97 (12)	2/69 (3)		
Cirrhosis	9/160 (6)	2/75 (3)		4/97 (4)	3/69 (4)		
**Non alcoholo-steato-hepatitis (Nash)**, *n (%)*							
No	57/160 (36)	39/75 (52)	0.0001	41/97 (42)	NA	NA	0.04
Borderline	75/160 (47)	27/75 (36)		21/97 (22)	NA		
Yes	28/160 (17)	9/75 (12)		35/97 (36)	NA		
**Steatosis**							
< 5%	10/160 (6)	24/75 (32)*	0.0001	19/97 (20)**	13/69 (19)	0.38	< 0.0001
5–32	56/160 (35)	16/75 (21)		22/97 (23)	27/69 (39)		
33–65	46/160 (29)	16/75 (21)		27/97 (28)	10/69 (14)		
66–100	48/160 (30)	19/75 (25)		29/97 (30)	19/60 (28)		
**Interval between biopsy and serum, ***median (95%CI), Range days*	0[0; 1] -87;+89	0[-4; 1] -538; +540	< 0.0001	0[0; 0] -24;+49	0[0; 0] -42; +40	0.90	0.07
**Biopsy quality***mean (se)*							
Sample size	20.1 (0.5)	19.3 (0.8)	0.33	17.7 (0.7)	21.4 (0.9)	0.001	0.002
Number of fragment	2.2 (0.2)	2.4 (0.2)	0.88	2.3 (0.2)	2.2 (0.2)	0.79	0.61
Number of portal tracts	16.4 (0.6)	15.5 (0.9)	0.28	13.6 (0.6)	17.1 (1.0)	0.008	0.01
**Serum biochemical markers**, *mean (se)*							
α_2_-macroglobulin (*g/L*)	1.70 (0.05)	1.70 (0.10)	0.74	1.72 (0.06)	1.76 (0.08)	0.47	0.50
ALT *(IU)*	72 (3)	63 (5)	0.10	79 (5)	84 (10)	0.69	0.35
AST *(IU)*^†^	50 (3)	44 (3)	0.10	45 (3)	52 (5)	0.05	0.006
Apolipoprotein A1(*g/L*)	1.47 (0.02)	1.45 (0.06)	0.98	1.59 (0.07)	1.53 (0.04)	0.80	0.06
GGT (*IU/L*)	111 (12)	108 (15)	0.83	132 (12)	158 (19)	0.16	0.04
Haptoglobin (*g/L*)	1.09 (0.04)	1.18 (0.11)	0.41	1.12 (0.10)	1.02 (0.05)	0.70	0.39
Total bilirubin (*μmol/L*)	11.4 (0.5)	22.1 (9.5)	0.35	12.6 (0.6)	13.6 (1.0)	0.89	0.02
Glucose *(mmol/L)*	6.1 (0.2)	5.3 (0.2)	0.08	6.1 (0.2)	5.8 (0.3)	0.008	0.46
Cholesterol *(mmol/L)*	5.5 (0.1)	5.5 (0.2)	0.87	5.4 (0.1)	5.5 (0.1)	0.57	0.84
Triglycerides *(mmol/L)*	2.0 (0.1)	1.6 (0.1)	0.31	1.4 (0.1)	1.3 (0.1)	0.03	0.04
FibroTest (*0.00–1.00*)	0.30 (0.02)	0.35 (0.05)	0.59	0.29 (0.02)	0.33 (0.02)	0.21	0.99
SteatoTest (*0.00–1.00*)	0.70 (0.01)	0.56 (0.05)	0.007	0.61 (0.03)	0.60 (0.03)	0.54	0.02

NA=Not applicable as patients excluded have other associated diseases

* = among the 24 patients excluded 20 had no steatosis and 4 had steatosis < 5%

**Table 2:** Univariate predictors of NASH in training and validation groups

Characteristic	Training Group				Validation Group			
	
	No NASH n = 57	Borderline NASH n = 75	NASH n = 28	P-value	No NASH n = 41	Borderline NASH n = 21	NASH n = 35	P-value
Demographics								
Age at biopsy, years	53.7 (1.5)	52.6 (1.3)	54.2 (2.1)	0.80	44.8 (1.8)	47.3 (2.8)	53.6 (2.1)	0.008
Male gender	35 (61%)	49 (65%)	12 (43%)	0.11	21 (51%)	17 (81%)	19 (54%)	0.06
Weight, kg	79.3 (1.9)	86.8 (1.69)	83.9 (2.8)	0.02	69.7 (2.0)	86.6 (2.78)	81.1 (2.15)	< 0.0001
Height, meter	1.69 (0.01)	1.68 (0.01)	1.66 (0.02)	0.54	1.67 (0.01)	1.74 (0.02)	1.68 (0.02)	0.02
Biochemical markers								
α_2_-macroglobulin, g/L	1.61 (0.09)	1.69 (0.08)	1.90 (0.13)	0.18	1.54 (0.10)	1.87 (0.14)	1.83 (0.11)	0.06
ALT, IU/L	61 (6)	78 (5)	81 (8)	0.03	75 (8)	60 (10)	94 (8)	0.03
AST, IU/L	42 (4)	53 (4)	57 (6)	0.06	37 (4)	32 (6)	62 (5)	< 0.0001
Apolipoprotein A1, g/L	1.54 (0.04)	1.41 (0.04)	1.49 (0.06)	0.08	1.71 (0.10)	1.48 (0.15)	1.51 (0.11)	0.32
Haptoglobin, g/L	1.08 (0.07)	1.14 (0.06)	1.01 (0.10)	0.52	1.24 (0.15)	1.02 (0.21)	1.04 (0.16)	0.56
GGT, IU/L	107 (19)	103 (17)	139 (28)	0.54	169 (18)	78 (26)	120 (20)	0.02
Total bilirubin, μmol/L	10.6 (0.9)	11.9 (0.7)	11.6 (1.2)	0.50	12.0 (0.89)	12.6 (1.24)	13.2 (0.96)	0.69
Glucose mmol/L	5.7 (0.3)	6.1 (0.3)	7.0 (0.4)	0.051	5.2 (0.31)	5.8 (0.44)	7.3 (0.34)	< 0.0001
Cholesterol mmol/L	5.5 (0.2)	5.4 (0.2)	5.5 (0.2)	0.98	5.4 (0.21)	5.62 (0.29)	5.25 (0.23)	0.61
Triglycerides	1.6 (0.2)	2.1 (0.2)	2.4 (0.4)	0.16	1.15 (0.11)	1.52 (0.16)	1.67 (0.12)	0.008
FibroTest	0.26 (0.03)	0.31 (0.02)	0.35 (0.04)	0.18	0.23 (0.03)	0.32 (0.04)	0.35 (0.03)	0.02
SteatoTest	0.64 (0.02)	0.72 (0.02)	0.75 (0.03)	0.007	0.54 (0.03)	0.61 (0.05)	0.69 (0.04)	0.02

All data are means (se) and proportions (n [%]). ALT, alanine aminotransferase; AST, aspartate aminotransferase; GGT, γ-glutamyl-transpeptidase.

**Table 3:** Univariate and multivariate predictors of NASH in the overall population (training and validation groups together)

Characteristic				No NASH vs Borderline or NASH			NASH vs Borderline or No NASH		
	
	No NASH n = 98	Borderline NASH n = 96	NASH n = 63	Univariate analysis P-value	Multivariate analysis P value	Odds Ratio (95% CI)	Univariate analysis P-value	Multivariate analysis P value	Odds Ratio (95% CI)
Demographics									
Age at biopsy, years	49.4 (1.2)	51.4 (1.2)	53.8 (1.5)	0.045	0.75	1.00 (0.97;1.04)	0.04	0.99	1.00 (0.97;1.33)
Male gender	56/98 (57%)	66/96 (69%)	31/63 (49%)	0.54	0.82	1.10 (0.48;2.56)	0.054	0.04	2.54 (1.03;6.26)
Weight, kg	75.2 (1.4)	86.8 (1.4)	82.3 (1.8)	< 0.0001	< 0.0001	1.06 (1.03;1.08)	0.25	0.86	1.00 (0.97;1.02)
Height, meter	1.68 (0.01)	1.69 (0.01)	1.67 (0.01)	0.87	0.15	0.03 (0.00;3.34)	0.22	0.66	0.33 (0.00;47.2)
Biochemical markers									
α_2_-macroglobulin, g/L	1.58 (0.07)	1.73 (0.07)	1.86 (0.08)	0.02	0.15	0.33 (0; 1050)	0.01	0.16	0.72 (0.46;1.14)
ALT, IU/L	67 (5)	74 (5)	88 (6)	0.005	0.90	1.00 (0.99;1.01)	0.004	0.24	0.99 (0.99;1.00)
AST, IU/L	40 (3)	48 (3)	60 (4)	0.0004	0.007	1.02 (1.01;1.04)	0.001	0.25	0.99 (0.98;1.01)
Apolipoprotein A1, g/L	1.61 (0.05)	1.43 (0.05)	1.50 (0.06)	0.08	0.27	0.53 (0.17;1.67)	0.62	0.71	1.14 (0.57;2.28)
Haptoglobin, g/L	1.14 (0.10)	1.11 (0.07)	1.02 (0.09)	0.87	0.50	0.83 (0.47;1.44)	0.41	0.18	1.56 (0.81;2.99)
GGT, IU/L	133 (14)	98 (14)	128 (17)	0.002	0.005	1.00 (0.993;0.998)	0.48	0.37	1.00 (0.998;1.00)
Total bilirubin, μmol/L	11.2 (0.6)	12.0 (0.6)	12.5 (0.8)	0.87	0.22	1.04 (0.98;1.09)	0.30	0.35	0.98 (0.93;1.02)
Glucose mmol/L	5.5 (0.2)	6.0 (0.2)	7.2 (0.3)	0.005	0.05	1.20 (0.999;1.45)	< 0.0001	0.0009	0.77 (0.67;0.90)
Cholesterol mmol/L	5.4 (0.1)	5.5 (0.1)	5.4 (0.2)	0.68	0.25	1.17 (0.90;1.53)	0.77	0.95	1.01 (0.78;1.30)
Triglycerides	1.4 (0.2)	2.0 (0.2)	2.0 (0.2)	< 0.0001	0.23	1.19 (0.90;1.57)	0.007	0.90	1.01 (0.83;1.24)
FibroTest	0.25 (0.02)	0.31 (0.01)	0.35 (0.02)	0.03	Not included	Not included	0.008	Not included	Not included
SteatoTest	0.60 (0.02)	0.70 (0.02)	0.72 (0.03)	< 0.0001	Not included	Not included	0.007	Not included	Not included

All data are means (se) and proportions (n [%]). ALT, alanine aminotransferase; AST, aspartate aminotransferase; GGT, γ-glutamyl-transpeptidase.

**Table 4:** Concordance between NASH predicted by NashTest and predicted by biopsy

**NashTest diagnosis**	**Histological Diagnosis**								
	**Training Group n = 160**			**Validation Group n = 97**			**All patients n = 257**		

	**No NASH**	**Borderline**	**NASH**	**No NASH**	**Borderline**	**NASH**	**No NASH**	**Borderline**	**NASH**

**No NASH**	20	7	0	29	4	8	49	11	8
**Borderline**	34	61	17	12	16	17	46	77	34
**NASH**	3	7	11	0	1	10	3	8	21
**Kappa Statistic**	0.28 ± 0.05			0.36 ± 0.06			0.33 ± 0.04		
Discordance one class	65/160 = 41%			34/97 = 35%			99/257 = 39%		
Discordance two classes	3/160 = 2%			8/97 = 9%			11/257 = 4%		

**Table 5:** Sensitivity, specificity and predictive values of NashTest for the diagnosis of NASH

**NashTest diagnosis**	**Histological Diagnosis**											
	**Training Group n = 160**				**Validation Group n = 97**				**All patients n = 257**			

	**Se**	**Sp**	**PPV **	**NPV**	**Sensitivity**	**Specificity**	**PPV **	**NPV**	**Sensitivity**	**Specificity**	**PPV **	**NPV**

**NASH**	11/28 39%	122/132 92%	11/21 52% Prevalence = 0.18	122/139 88%	10/35 29%	61/62 98%	10/11 91% Prevalence = 0.36	61/86 71%	21/63 33%	183/194 94%	21/32 66% Prevalence = 0.25	183/225 81%
**Borderline or NASH**	96/103 93%	20/57 35%	96/133 72% Prevalence = 0.64	20/27 74%	44/56 79%	29/41 71%	44/56 79% Prevalence = 0.58	29/41 71%	140/159 88%	49/58 50%	140/189 74% Prevalence = 0.62	49/68 72%

**Table 6:** Sensitivity analyses of NashTest for the diagnosis of Nash versus No Nash or Borderline Nash [Area under the ROC curves (AUROCs)], according to group, ALT values, alcohol consumption, quality of biopsy, inclusion of patients without steatosis, and exclusion of high risk of biomarkers failures.

**Characteristic of patients**	**n**	**NashTest AUROC**	**Lower 95% **	**Upper 95%**	**Prevalence NASH**	**Significance between AUROCs**
**All 257 patients**	257	0.75	0.67	0.82	0.25	
Stratified according to Group*						
Training Group	160	0.79	0.69	0.86	0.18	0.94
Validation Group	97	0.79	0.67	0.87	0.36	
Stratified according to ALT value*						
ALT < 50 IU/L	92	0.78	0.58	0.89	0.17	0.58
ALT >= 50 IU/L	165	0.73	0.64	0.81	0.28	
Stratified according to Alcohol consumption*						
None	95	0.72	0.57	0.82	0.31	0.78 and 0.28
< 30 g for men and < 20 g for female	150	0.77	0.66	0.84	0.19	0.78 and 0.33
>= 30 g for men or >= 20 g for female	12	0.94	0.53	0.99	0.50	0.28 and 0.33
Quality of biopsy*						
< 25 mm	194	0.76	0.66	0.83	0.25	0.72
>= 25 mm	63	0.73	0.55	0.84	0.24	
Fragmented	148	0.71	0.58	0.80	0.22	0.15
Not Fragmented	109	0.81	0.70	0.88	0.28	
**Patients included or excluded**						
Patients without steatosis included**	277	0.76	0.68	0.82	0.39	0.85
High risk of NashTest failures excluded**						
Gilbert excluded	244	0.76	0.68	0.82	0.25	0.78
Acute Inflammation excluded	254	0.75	0.67	0.82	0.24	0.95

* Comparison between stratified groups

** Comparison with all 257 patients

**Table 7:** Sensitivity analyses of NashTest for the diagnosis of Borderline Nash or Nash versus No Nash [Area under the ROC curves (AUROCs)], according to group, ALT values, alcohol consumption, quality of biopsy, inclusion of patients without steatosis, and exclusion of high risk of biomarkers failures.

**Characteristic of patients**	**n**	**NashTest AUROC**	**Lower 95% **	**Upper 95%**	**Prevalence Borderline or NASH**	**Significance**
**All 257 patients **	257	0.78	0.72	0.84	0.38	
Stratified according to Group*						
Training Group	160	0.77	0.68	0.84	0.36	0.34
Validation Group	97	0.83	0.67	0.90	0.42	
Stratified according to ALT value*						
ALT< 50IU/L	92	0.74	0.62	0.82	0.50	0.24
ALT>= 50 IU/L	165	0.81	0.73	0.87	0.32	
Stratified according to Alcohol consumption*						
None	95	0.78	0.67	0.86	0.37	0.83 and 0.20
< 30 g for men and < 20 g for female	150	0.77	0.66	0.84	0.39	0.83 and 0.19
>= 30 g for men or >= 20 g for female	12	1.00	0.35	1.00	0.33	0.20 and 0.19
Stratified according to Quality of biopsy*						
< 25 mm	194	0.78	0.71	0.84	0.39	0.98
>= 25 mm	63	0.78	0.63	0.88	0.37	
Fragmented	148	0.77	0.68	0.84	0.36	0.46
Not Fragmented	109	0.81	0.71	0.88	0.40	
**Patients included or excluded**						
Patients without steatosis included**	272	0.79	0.72	0.84	0.39	0.83
High risk of NashTest failures excluded**						
Gilbert excluded	244	0.78	0.71	0.83	0.39	0.97
Acute Inflammation excluded	254	0.78	0.72	0.84	0.38	0.98

* Comparison between stratified groups

** Comparison with all 257 patients
